# Anomalous Origination of Right Coronary Artery from Left Sinus in Asymptomatic Young Male Presenting with Positive Ischemic Response on Treadmill Test

**DOI:** 10.1155/2016/7652869

**Published:** 2016-01-14

**Authors:** Budi Yuli Setianto, Anggoro Budi Hartopo, Putrika Prastuti Ratna Gharini, Nahar Taufiq

**Affiliations:** Department of Cardiology and Vascular Medicine, Faculty of Medicine, Universitas Gadjah Mada and Dr. Sardjito Hospital, Yogyakarta 55281, Indonesia

## Abstract

Anomalous origination of coronary artery from the opposite sinus (ACAOS) is a rare coronary artery anomaly. Right ACAOS with interarterial course is a type of ACAOS, which conveys a high risk for myocardial ischemia or sudden death. We reported a case of right ACAOS with interarterial course in otherwise healthy young male. He was asymptomatic, until an obligatory medical check-up with treadmill test showed a sign of positive ischemic response. Further work-up revealed that he had right ACAOS with interarterial course. Watchful observation was applied to him, while strenuous physical activity and competitive sport were absolutely prohibited.

## 1. Introduction

Anomalous origination of coronary artery from the opposite sinus (ACAOS) is an uncommon coronary anomaly. Its incidence is reported to be around 1.07% [1]. It comprises anomaly of right coronary artery originated from left sinus (right ACAOS) and its opposite or left ACAOS. The incidence of right ACAOS is between 0.12% and 0.92% [1, 2]. Both right and left ACAOS have significant clinical consequence if the ectopic artery has an interarterial course or intramural intussusception [1]. Myocardial ischemia is clinical symptoms and signs frequently associated with ACAOS with interarterial course. A constant relationship is observed between left ACAOS and sudden death or ischemia during extreme exercise [1]. Right ACAOS with an interarterial course is a type of ACAOS which poses high risk for myocardial ischemia or sudden death as well [3]. However, most ACAOS does not reveal signs and symptoms; therefore the diagnosis is often found in postmortem autopsy. We described a case of asymptomatic young male who underwent treadmill test for obligatory medical check-up and the result showed a positive ischemic response. Further investigation revealed that he suffered from right ACAOS with an interarterial course.

## 2. Case Report

**Figure 1:**
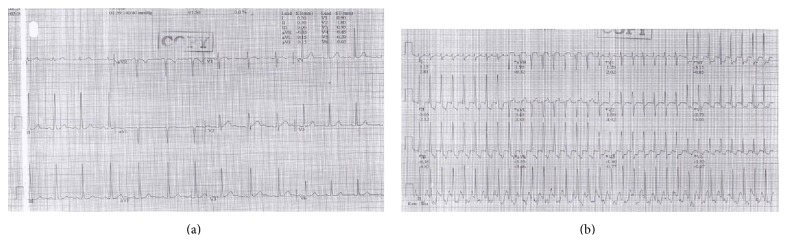
Electrocardiogram of the case before treadmill test (a) and during treadmill test showing ischemic sign (b).

A 28-year-old male was referred to cardiology unit of our hospital from a general practitioner due to positive ischemic response on treadmill test during his obligatory medical check-up. He underwent medical check-up as an obligation related to his career. The result of Bruce-method treadmill test was positive ischemic response, good physical fitness, and aerobic capacity of 14.37 Mets (Figure 1). During treadmill test, the patient did not complain of chest pain; however his electrocardiogram showed horizontal ST depression indicating myocardial ischemia. Anamnesis revealed no history of chest pain, dyspnea on effort, dyspnea at rest, syncope, palpitation at rest, and palpitation on activity. The patient had no family history of sudden death or similar abnormality. Physical examination was within normal limit. Laboratory examination showed normal value. Electrocardiogram at rest indicated sinus rhythm without sign of ischemia (Figure 1).

A noninvasive examination was performed with echocardiography. Transthorax echocardiogram showed normal cardiac chamber dimension, normal right ventricle and left ventricle wall thickness, normal left and right ventricle systolic and diastolic function, and normal left ventricular segmental and global wall motion. Mitral valve and tricuspid valve were anatomically and functionally normal. Aortic valve examination showed three cusps with normal anatomy and function. Ostium of left coronary artery (LCA) was apparent with diameter of 5 mm, whereas ostium of right coronary artery (RCA) was absent.

**Figure 2:**
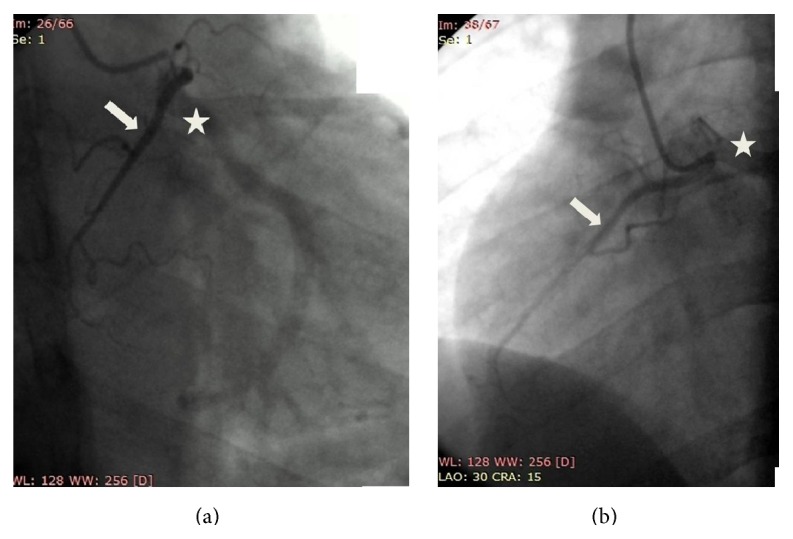
(a) and (b) Coronary angiography (RAO 20 Caudal 20 view and LAO 30 Cranial 15 view) shows RCA (arrow) originated from left sinus and its ostium was adjacent to LCA ostium (star). The caliber of RCA was small and coursed into right ventricles with normal bifurcation.

Coronary angiography was performed for the patient, started by cannulation into ostium of LCA with Tiger 6 F catheter via radial access. An LCAgraph by contrast agent showed normal left main (LM), normal left anterior descendent (LAD), and left circumflexus (LCx). On LCAgraph view of RAO 20 Caudal 20 and LAO 30 Cranial 15, an RCA was originated from left sinus near LM and coursed through the right aspect of heart (Figure 2). The caliber of RCA was small with normal bifurcation. Posterior descending artery was filled from LCx (left dominance).

**Figure 3:**
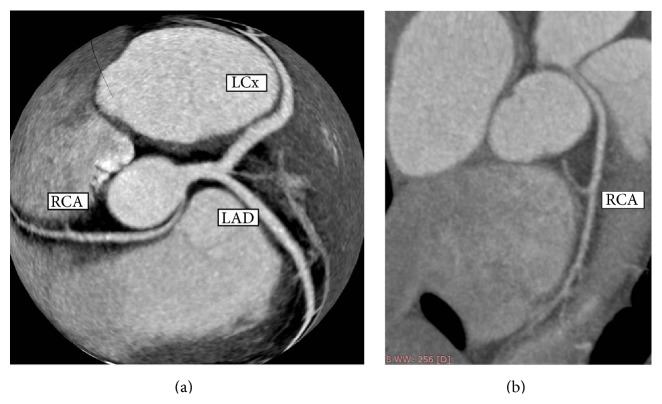
(a) and (b) Cardiac MSCT shows RCA originated from left sinus of Valsalva adjacent to LCA ostium with interarterial course between ascending aorta and pulmonary artery. The caliber of RCA was small and coursed into right atrioventricular sulcus.

**Figure 4:**
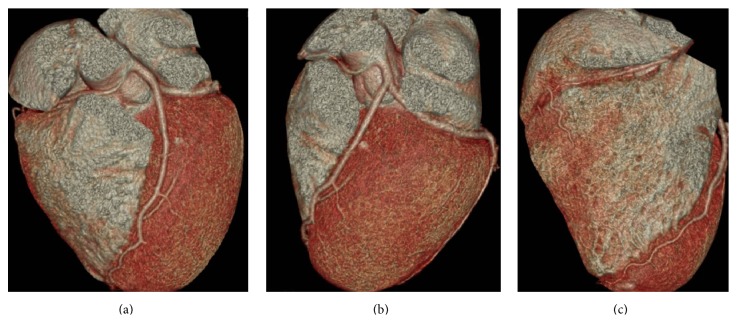
(a)–(c) Reconstruction 3D cardiac MSCT shows RCA originated from left sinus of Valsalva adjacent to LCA ostium with interarterial course between ascending aorta and pulmonary artery through right atrioventricular sulcus.

The confirmation by cardiac multi slice CT (cMSCT) scan showed that RCA originated from left sinus of Valsalva with interarterial course between ascending aorta and pulmonary artery. The caliber of RCA was small with no sign of stenosis in the RCA (Figures 3 and 4). The small RCA runs through right atrioventricular sulcus and vascularized the right part of the heart. The proximal part of RCA passed through ascending aorta and pulmonary artery.

Based on the diagnostic work-up, the patient was diagnosed as right ACAOS with interarterial course. The evidence of myocardial ischemia, as depicted by positive ischemic response on treadmill test, was evident in the exercise electrocardiogram of this patient although asymptomatic. Right ACAOS with interarterial course was responsible for the ischemia sign. No other structural cardiac abnormalities were found in the patient; therefore the coronary anomaly was the most likely etiology of the ischemia sign. After in-depth consultation about the prognostic implication of this anomaly, the patient and family decided that surgical correction was not to be done then. Therefore, in this patient, conservative management was employed. Watchful observation was applied to the patient, while strenuous physical activity and competitive sport were absolutely prohibited. Beta blocker as needed was given to the patient. During eight-month follow-up after the diagnosis, the patient reported no ischemic symptom during regular activity. He never took beta blocker medication then.

## 3. Discussion

We report a case of 28-year-old male who suffered from right ACAOS with interarterial course and the sign of myocardial ischemia. Close observation and limitation of strenuous activity were advised. Neither medication nor surgical revascularization was applied to the patient. Eight-month follow-up of this patient was uneventful.

Coronary artery anomaly is an uncommon condition with prevalence on coronary angiography between 0.61% and 5.64% [2]. The incidence of right ACAOS from coronary angiography is between 0.12% and 0.92% [1, 2]. Right ACAOS with interarterial course is a group of ACAOS with high risk for developing myocardial ischemia and sudden death [3]. Close and reliable relationship is observed between left ACAOS and the incidence of sudden death and ischemia during strenuous physical activity [1]. The clinical picture of ACAOS can be divided into two spectra: the first is sudden death in the young and after strenuous physical activity or sport and the second is atypical clinical picture [1]. Most of ACAOS patients are asymptomatic. Atypical chest discomfort is the most prevalent symptom urging patients to refer to the health facility and to perform the coronary angiography to detect ACAOS. Some patients come due to positive stress test or sign of ischemic heart disease on ECG [1]. In this case, patient was referred due to positive stress test without any ischemic symptoms previously.

Clinical implication of coronary artery anomaly can be divided into ischemic and nonischemic. Ischemic implication can be fixed or episodic ischemia [1]. Right ACAOS with interarterial course is associated with episodic myocardial ischemia. Interarterial course means that ectopic coronary artery runs through two big vessels arising from ventricle, that is, aorta and pulmonary artery. Three mechanisms are proposed regarding the proneness of right ACAOS with interarterial course to develop ischemia or sudden death, that is, sharp angulation and kinking of coronary artery while running off from the opposite sinus, valve-like mechanism causing acute closure in the slit-like coronary artery ostium, and compression of narrowed segment of coronary artery by aorta or pulmonary artery particularly during strenuous activity [4]. Hard activity causes dilatation of aortic root and pulmonary trunk which compresses slit-like ostium or particular segment of ectopic coronary artery. This occurs especially in individuals with sufficient aortic distensibility, such as in young people or sportsmen [1]. In our case, the patient was still young and without any complaints during daily activity and regular excercise. Ischemic sign appeared in the treadmill test marked by down-slopping and horizontal ST-depression in stage 4 Bruce-method treadmill test. We speculated that compression of RCA by big vessels was responsible for the ischemic sign.

However, the unusual thing about this case is the fact that the RCA was not a dominant vessel because, based on angiogram and cMSCT, the PDA aroused from LCx. In most cases reported with ischemic symptoms associated with right ACAOS, the RCA is a dominant vessel; therefore obstruction of this vessel produces significant myocardial ischemia. Echocardiogram of the patient showed no other signs of structural cardiac abnormality; therefore the most likely source of ischemia was intramural course of the RCA anomaly. The left dominance nature of the patient prevented, so far, fatal ischemia. Furthermore, the caliber of anomalous RCA, which was small, may also account for the significant ischemic ECG changes developed during excercise test.

Treatment modalities for right ACAOS with signs and symptoms of ischemia are watchful observation and drugs and coronary angioplasty with stent and corrective surgery [5]. The goal of right ACAOS treatment is to prevent sudden death and improve quality of life [3]. In this patient, after being given several alternatives treatment modalities, watchful observation was selected by the patient and his family. Restriction of strenuous activity and competitive sport was encouraged since these may cause ischemic myocardial or sudden death. Maron and Zipes (2005) stated that right ACAOS patients without intervention should not involve in competitive sport and hard physical activity [6]. The report of close observation and medication with beta blocker within 2 and 5 years in ACAOS results in zero sudden death [7]. The ACC/AHA guideline in 2008 stated that conservative approach is reasonable in right ACAOS without evidence of ischemia [8]. However, since in this patient the evidence of ischemia was present, surgical coronary revascularization should be performed (level of evidence B) [8].

## 4. Conclusion

In conclusion, this is a case report of rare coronary artery anomaly, right ACAOS with interarterial course, with evidence of ischemic sign, that is, positive ischemic response in treadmill test, in otherwise asymptomatic young male. Wacthful observation and strenuous activity restriction were applied in this case.

## References

[1] Angelini P. (2007). Coronary artery anomalies: an entity in search of an identity. *Circulation*.

[2] Yurtdas M., Gülen O. (2012). Anomalous origin of the right coronary artery from the left anterior descending artery: review of the literature. *Cardiology Journal*.

[3] Barriales-Villa R., Tassa C. M. (2006). Congenital coronary artery anomalies with origin in the contralateral sinus of valsalva: which approach should we take?. *Revista Espanola de Cardiologia*.

[4] Tsujita K., Maehara A., Mintz G. S. (2009). In vivo intravascular ultrasonic assessment of anomalous right coronary artery arising from left coronary sinus. *The American Journal of Cardiology*.

[5] Angelini P. (2002). Coronary artery anomalies—current clinical issues: definitions, classification, incidence, clinical relevance, and treatment guidelines. *Texas Heart Institute Journal*.

[6] Maron B. J., Zipes D. P. (2005). Introduction: eligibility recommendations for competitive athletes with cardiovascular abnormalities—general considerations. *Journal of the American College of Cardiology*.

[7] Bixby M. B. (1998). Successful medical management of a patient with an anomalous right coronary artery who declined surgery. *American Journal of Critical Care*.

[8] Warnes C. A., Williams R. G., Bashore T. M. (2008). ACC/AHA Guidelines for the management of adults with congenital heart disease: a report of the American College of Cardiology/American Heart Association Task Force on Practice Guidelines (Writing Committee to Develop Guidelines for the Management of Adults With Congenital Heart Disease). *Circulation*.

